# Interlaboratory study to assess precision and reproducibility of the meningococcal antigen surface expression (MEASURE) assay to quantify factor H binding protein expression at the surface of meningococcal serogroup B strains

**DOI:** 10.1016/j.diagmicrobio.2025.116920

**Published:** 2025-05-30

**Authors:** Jakob Loschko, Paul Liberator, Jamie Findlow, Jason Yip, Charles Tan, Karen Garcia, MaryAnn Murillo, Yamini Gorantla, Kimberly M. Moss, Panagiotis Maniatis, Stephen A. Clark, Ray Borrow

**Affiliations:** a Vaccines Research & Development, Pfizer Inc, 401 N Middletown Rd, Pearl River, NY 10965, USA; b Vaccines/Antivirals and Evidence Generation, Pfizer Ltd, Walton Oaks, Dorking Road, Tadworth, Surrey, KT20 7NS, UK; c Early Clinical Development, Pfizer Inc, 401 N Middletown Rd, Pearl River, NY 10965, USA; d IHRC, 2 Ravinia Dr NE, Atlanta, GA 30346, USA; e Eagle Global Scientific, 2835 Brandywine Rd, Atlanta, GA 30341, USA; f Centers for Disease Control and Prevention, 1600 Clifton Road NE, Atlanta, GA 30329, USA; g Meningococcal Reference Unit, UK Health Security Agency, Manchester Royal Infirmary, Oxford Rd, Manchester, M13 9WL, UK

**Keywords:** Factor H binding protein, MEASURE assay, Meningococcal, Precision, Reproducibility, Surface expression

## Abstract

**Background:**

The serum bactericidal antibody using human complement (hSBA) assay, the accepted surrogate measure of meningococcal vaccine efficacy, is limited by human sera and complement requirements. Pfizer developed and validated the flow-cytometry–based Meningococcal Antigen Surface Expression (MEASURE) assay to quantify surface-expressed factor H binding protein (fHbp) levels on intact meningococci. Surface expression of fHbp is correlated with hSBA assay killing by MenB-fHbp (Trumenba^®^)–induced antibody, meaning the MEASURE assay can be used to predict meningococcal serogroup B (MenB) strain susceptibility to antibodies elicited by MenB-fHbp. This study aimed to evaluate interlaboratory precision and reproducibility of the MEASURE assay.

**Methods:**

The MEASURE assay was transferred to UK Health Security Agency (UKHSA) and US Centers for Disease Control and Prevention (CDC) laboratories. MEASURE assay results from 42 MenB strains encoding sequence-diverse fHbp variants that express fHbp at different levels were compared between the UKHSA, CDC, and Pfizer laboratories. Intermediate precision within each laboratory was determined.

**Results:**

Pairwise comparisons of fHbp expression levels for all 42 MenB test strains showed >97 % agreement across the 3 laboratories when strains were grouped above or below a mean fluorescence intensity level of 1000, the threshold previously established as indicative of susceptibility to MenB-fHbp–induced antibodies in the hSBA assay. Each laboratory met assay precision criteria of ≤30 % total relative standard deviation.

**Conclusions:**

Quantification of fHbp surface expression using the MEASURE assay is robust and reproducible across different laboratories. Previously determined cutoffs corresponding to predicted susceptibility to vaccine-induced antibodies can be applied to MEASURE data generated across laboratories.

## 1. Introduction

Invasive meningococcal disease (IMD) is a relatively rare disease with potentially severe consequences, including death (case fatality rate, 8.3 % in a recent systematic review [1]) or severe sequelae (11 %–19 % of survivors), such as limb amputation or neurologic deficits [2]. Although relative incidence rates across age groups are geographically variable, those at greatest risk of IMD include infants and young children, adolescents and young adults, and older adults [3]. Meningococcal serogroups A, B, C, W, and Y have caused the vast majority of IMD in recent years, with serogroup B (MenB) causing nearly half of all IMD globally [3,4]. Additionally, several serogroup X outbreaks have occurred in the African meningitis belt countries, with sporadic cases observed in other regions [5].

The rapid progression characteristic of IMD [6] coupled with its potential for severe outcomes indicate prophylactic vaccination as the best method of averting the adverse effects of IMD [7]. Because the capsular polysaccharide is required for survival of the meningococcus during invasive disease in immunocompetent hosts [8], polysaccharide-based vaccines have been successful in preventing IMD caused by serogroups A, C, W, and Y [9]. However, development of MenB polysaccharide-based vaccines has been impeded by poor vaccine immunogenicity and concern regarding autoimmune effects [10,11], ultimately leading to the development of surface protein vaccines [7,12].

Because of the relative rarity of IMD, efficacy trials for meningococcal vaccines would require impractically large numbers of study participants [13]. Instead, bactericidal antibody in human sera is quantified using the serum bactericidal antibody (SBA) assay, which is considered an acceptable surrogate measure of protection to support meningococcal vaccine licensure in the absence of efficacy data [13,14]. Meningococcal serogroups A, C, W, and Y (MenACWY) polysaccharide-based conjugate vaccines have been licensed on the basis of SBA assay data obtained using human (hSBA) or baby rabbit complement [15]. These SBA assays use a single indicator strain for each serogroup to determine functional immune responses [15,16]. Because the same essential polysaccharide is expressed by all strains with the corresponding capsule, the data generated in the assay against a single strain are considered transferable to all other strains belonging to the same serogroup [16,17].

Unlike the ability of MenACWY polysaccharide-based vaccines to protect across all strains with the corresponding capsule, current MenB surface protein vaccines are not expected to cover all MenB strains [16]. A series of factors contributes to the ability of the immune response induced by protein-based vaccines to protect against diverse, disease-causing MenB strains; these include the presence or absence of the gene coding for the protein, the level of surface expression of the protein, and the similarity of the expressed protein variant harbored by the strain to the vaccine antigen [16]. Understanding the breadth of coverage across diverse MenB strains afforded by a particular surface protein antigen or vaccine formulation is therefore required [16]. One potential approach to provide detailed breadth of coverage data could be the testing of vaccine-induced SBA against large numbers of diverse MenB strains [16]. However, this is not practical for hSBA assays due to difficulties in obtaining human complement, which is the only suitable source of complement for MenB SBA assays [13,14], and vaccinee serum volume limitations [16,18], as well as the practicalities of testing large numbers of strains.

Both of the currently licensed MenB vaccines, MenB-fHbp (Trumenba^®^; Pfizer Inc, Philadelphia, PA, USA) and MenB-4C (Bexsero^®^; GSK Vaccines, Srl, Sovicille, Italy), include at least one factor H binding protein (fHbp) antigen [19,20]. fHbp is a surface-exposed meningococcal protein that downregulates complement activity, facilitating bacterial survival in human blood [21–23]. With few exceptions, the gene coding for fHbp is present in all disease-causing MenB strains [24], with fHbp almost ubiquitously expressed and highly immunogenic; additionally, fHbp protein variants segregate into 2 subfamilies based on variations in amino acid sequence [25,26]. Further evaluations for MenB-fHbp demonstrated that, compared with nonlipidated fHbp antigens, lipidated fHbp antigens induced greater levels of SBA and showed increased functional cross-reactivity to MenB strains expressing vaccine-heterologous fHbp variants [26,27]. Because bactericidal activity induced by a given lipidated fHbp antigen was largely limited to strains harboring fHbp variants within the same genetically defined subfamily [26], MenB-fHbp is formulated as a bivalent vaccine containing 2 lipidated fHbp variants, 1 from each of the 2 subfamilies, to provide broad protection against MenB strains [20,28]. The immune-enhancing effects of fHbp lipidation have led MenB-fHbp to be described as a self-adjuvanting vaccine [29]. In contrast with MenB-fHbp, MenB-4C includes a single, non-lipidated, subfamily B fHbp variant, presented as a recombinant fusion protein with GNA2091 to enhance the immunogenicity of the antigen [17,19]. Based on this difference as well as the inclusion of additional antigens in MenB-4C [19], the immunogenicity and predicted strain coverage are not necessarily comparable between the 2 vaccines [17].

Bacterial genome sequence data have uncovered a large diversity of fHbp variants (>1400 peptide variants as of June 2023) [30], which renders the evaluation of strains with each variant in hSBA assays impractical [16]. Breadth of coverage assessments for the MenB surface protein vaccines must therefore rely on hSBA data from test strains that accurately represent the diversity of MenB isolates [16,27,31]. For MenB-fHbp, immunogenicity assessments have relied on hSBA data from a panel of 4 primary and 10 additional MenB test strains, each expressing a diverse fHbp variant different from the vaccine antigens [27,31]. These strains were carefully selected to be representative of the breadth of meningococcal fHbp diversity [27,31]. Robust immune responses against these strains were observed in MenB-fHbp clinical studies, demonstrating vaccine-induced cross-reactive antibodies irrespective of the fHbp variant expressed by the MenB test strains and supporting broad coverage of MenB-fHbp across the diversity of MenB strains [17,32].

In addition to sequence diversity, selection criteria for the 14 MenB test strains included low to medium levels of fHbp expression [27,31]. However, fHbp surface expression varies by >100-fold [18], and strains may not be covered by MenB-fHbp if their fHbp expression is too low to enable sufficient binding of vaccine-induced SBA to result in bacterial lysis [17,18]. Pfizer therefore developed the flow-cytometry–based Meningococcal Antigen Surface Expression (MEASURE) assay as a reliable method of accurately quantifying fHbp surface expression to establish a predictive hSBA killing threshold [18]. Specifically, the assay was used to evaluate a collection of 1814 globally representative MenB isolates that code for sequence-diverse fHbp variants collected by public health laboratories between 2000 and 2006 [18]. The susceptibility of 109 MenB isolates to MenB-fHbp–induced antibodies was tested in hSBA assays using sera from individuals vaccinated with MenB-fHbp and compared with fHbp expression levels [18]. Findings indicated that an fHbp expression level corresponding to a readout of >1000 mean fluorescence intensity (MFI) in the MEASURE assay was associated with a 91.2 % probability of being killed in the hSBA assay [18]. Within the larger strain pool, 91.4 % of isolates expressed fHbp at or above this threshold, indicating broad coverage of MenB-fHbp against diverse MenB isolates [18]. These results were further supported by MEASURE assays performed with MenB disease–causing isolates from Canada (2010–2012) and Greece (2010–2017), in which 91.2 % of 102 and 95.5 % of 66 isolates had fHbp expression levels >1000 MFI, respectively [33,34]. These findings demonstrate that despite different meningococcal genotypes and types of fHbp variant expressed, fHbp expression is stable over time and across geographic regions, possibly attesting to the important role that fHbp plays in bacterial survival.

Given the potential of the MEASURE assay to predict the susceptibility of MenB strains to MenB-fHbp–induced antibodies [18], Pfizer (Pfizer Vaccines Research & Development, Pearl River, NY, USA) transferred the assay to reference laboratories at the UK Health Security Agency (UKHSA; Meningococcal Reference Unit, Manchester, UK) and the US Centers for Disease Control and Prevention (CDC; Microbial Pathogenesis and Immune Response Laboratory, Atlanta, GA, USA) to support global meningococcal surveillance. Here, we report results from an interlaboratory study that was conducted to ensure that results are consistent and reproducible across the 3 laboratories.

## 2. Materials and Methods

### 2.1. MEASURE assay protocol and bacterial growth

Factor H binding protein surface expression was evaluated by the flow-cytometry–based MEASURE assay using a protocol that generally followed the approach published previously [35]. In brief, glycerol stocks were cultured on GC agar plates overnight for 16 to 18 hours at 37°C with 5 % CO_2_ without humidification. The following day, 250 to 2000 bacterial colonies, depending on colony size, were added to 25 mL GC medium base with Kellogg’s supplement and NaHCO_3_ to attain a starting A_650_ between 0.15 and 0.20 and incubated at 37°C (shaking at 150 rpm) until bacterial growth reached an A_650_ of 0.50 to 0.55. Subsequently, bacteria were collected by centrifugation at ambient temperature, resuspended in 5 mL of 1 % paraformaldehyde in phosphate-buffered saline, and fixed overnight (16‒24 hours) at 4°C. On the following day, cells were stained with MN86–994-11–1, a primary anti-fHbp broadly cross-reactive monoclonal antibody (mAb), or with an isotype control mouse mAb, followed by biotinylated anti-mouse immunoglobulin G antibody and then Streptavidin-Phycoerythrin (PE); each of these 3 steps was conducted for 30 minutes on ice or under chilled conditions with 2 washes between each step. Finally, samples were analyzed on a BD Accuri C6+ or BD FACSVia flow cytometer (BD Biosciences, San Jose, CA, USA).

### 2.2. MenB strains

**Figure 1.**
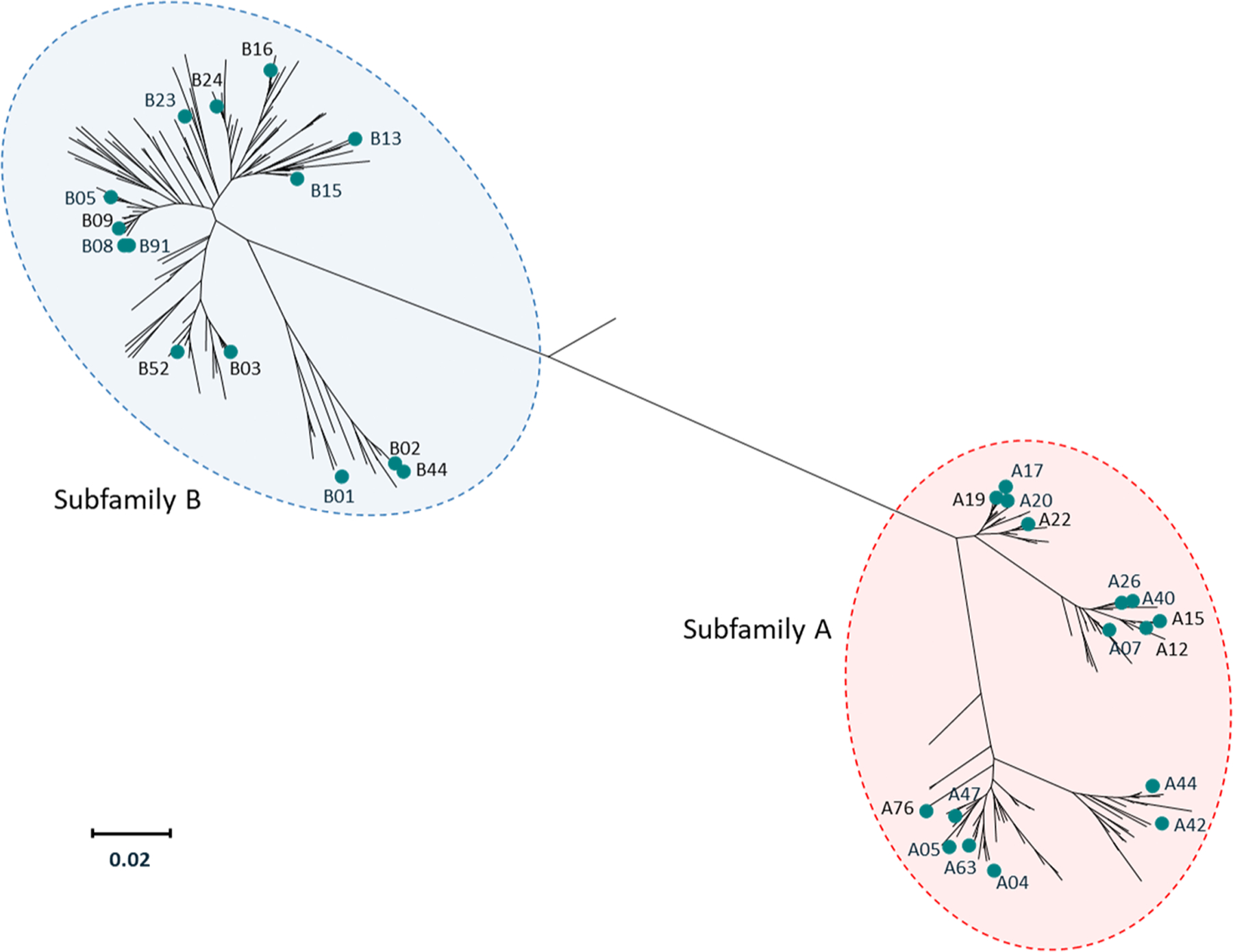
Factor H binding protein phylogenetic tree illustrating the sequence diversity of the fHbp variants expressed by the 42 MenB strains included in the interlaboratory study. The phylogenetic tree of fHbp variants encoded by strains from a multinational collection of MenB strains [36] is based on ClustalW alignment and drawn with MEGA 4. The end of each branch on the tree represents a specific fHbp variant, and the scale bar illustrates phylogenetic distance based on protein sequence. The teal circles illustrate the positions of the 30 fHbp variants expressed by the 42 strains evaluated in the interlaboratory study. These include 16 subfamily A fHbp variants (23 subfamily A strains in total) and 14 subfamily B fHbp variants (19 subfamily B strains in total). fHbp = factor H binding protein; MenB = meningococcal serogroup B.

Meningococcal serogroup B test strain selection from a multinational collection of invasive disease isolates [36] was based on the primary criteria of sequence diversity and inclusion of low, medium, and high fHbp-expressing strains. To achieve fHbp phylogenetic diversity, 22 test strains expressing 16 subfamily A and 18 test strains expressing 13 subfamily B fHbp variants were selected, for a total of 40 test strains; 2 additional strains, PMB1135 (fHbp variant B01) and PMB1745 (fHbp variant A05), were included as controls (Fig. 1). Table 1 lists all 42 strains along with corresponding fHbp expression levels that were determined at Pfizer using the MEASURE assay before the current interlaboratory study. The 16 subfamily A and 14 subfamily B fHbp variants collectively expressed by the group of 42 strains correspond to variants encoded by at least 79 % of invasive MenB isolates in collections from the United States (*N* = 442; collected during 2000‒2008) and Europe (*N* = 1052; 2007‒2008) [37,38]. These findings are similar to an earlier study in which genes coding for a subset of 10 fHbp variants accounted for 79.3 % of 1263 MenB strains collected in the United States (*N* = 432 collected during 2000‒2005) and Europe (*N* = 831; 2001‒2006) [39].

### 2.3. Interlaboratory study design

To evaluate interlaboratory reproducibility of MEASURE assay results, Pfizer prepared master stocks of the 42 MenB strains and then transferred aliquots to the CDC and UKHSA, where working stocks were prepared. Common reagent lots and equipment were used across all 3 laboratories. At each laboratory, the 42 strains were tested using a total of 4 plates, with each plate including 10 test strains and the 2 control strains; evaluation of fHbp expression for the group of 42 strains using 4 plates as outlined is considered an assay run. Four replicate wells of bacteria were prepared for analysis on each plate for each included strain, with 2 wells stained with the fHbp mAb MN86–994-11–1 and 2 wells stained with the isotype control mAb. Each lab tested the composite group of 42 strains twice. At the Pfizer and CDC laboratories, each of the 2 assay runs was performed by an independent analyst, whereas a single analyst at the UKHSA laboratory collected fHbp expression data for the 42 strains in 2 assay runs, each conducted during a different week (Supplemental Figure S1). Each laboratory thus generated 4 replicate data points using the fHbp mAb MN86–994-11–1 for each of the 40 test strains and 16 replicate data points for each of the 2 control strains.

Because the MFI output of flow cytometers can vary, the effects of a normalization step on assay precision as well as comparability of results generated at different labs were also evaluated. MFI results were therefore normalized to data from PE-labeled polystyrene beads that were measured on every assay day at each of the laboratories. PE-labeled polystyrene beads were tested prior to every run to ensure readouts fell within a pre-defined range; if the MFI from a cytometer fell outside this range, voltages were adjusted and/or the cytometer was cleaned.

### 2.4. Data analysis

Within each laboratory, the geometric mean MFI value for all replicates (ie, 4 for each of the test strains; 16 for each of the control strains) was calculated for each strain; geometric mean values for each strain were then compared across the 3 laboratories. Per previously established limits of detection [18,35], fHbp expression was defined as MFI ≥100 and >3 times the MFI measured using the non-specific isotype control antibody. For the comparisons, the assay data were separated into 2 groups based on fHbp expression levels corresponding to MEASURE readouts of ≤1000 or >1000 MFI, ie, the MFI threshold previously established to correspond to predominant susceptibility to MenB-fHbp–induced antibodies in the hSBA assay [18]. Pairwise interlaboratory comparisons were evaluated for each strain based on whether the strain expressed fHbp at levels ≤1000 or >1000 MFI at each of 2 different laboratories.

Variance components analysis (VCA) was then used to determine the intermediate precision of the MEASURE assay, with the estimated variance, standard deviation (log_10_), percentage of total variance, and percentage of relative standard deviation (%RSD) evaluated for each variance component. The prespecified precision acceptance target was total %RSD ≤30 %. Variance components included week (UKHSA) or analyst (Pfizer, CDC), nested plate [week] (UKHSA) or nested plate [analyst] (Pfizer, CDC), and residual (each laboratory). The “week” variance component was used for UKHSA data, which were collected by a single analyst over 2 runs performed on separate weeks. The “analyst” variance component was used for Pfizer and CDC data because the 2 assay runs were each conducted by an independent analyst.

For the Pfizer and CDC laboratories, the statistical VCA model used to relate the log-transformed geometric mean MFI to the experimental design, adjusting for sample effect, was

$$Y_{ijk}=\mu +\delta_{i}+A_{j}+R_{k(j)}+\varepsilon_{n(ijk)}$$

where Y_ijk_ is the log-transformed geometric mean MFI, μ is the overall mean, δ_*i*_ is the strain i effect, A_*j*_ is the analyst j effect, R_*k(j)*_ is the plate within each level of analyst k(j) effect (ie, nested plate [analyst]), and *ε*_*n(ijk)*_ is the residual. For the UKHSA laboratory, the statistical VCA model was

$$Y_{ijk}=\mu +\delta_{i}+W_{j}+R_{k(j)}+\varepsilon_{n(ijk)}$$

where Y_ijk_ is the log-transformed geometric mean MFI, μ is the overall mean, δ_*i*_ is the strain i effect, W_*j*_ is the week j effect, R_*k(j)*_ is the plate within each level of week k(j) effect (ie, nested plate [week]), and ε_*n(ijk)*_ is the residual. For both models, variability due to plate readers and within-week repeatability is captured in the residual variability.

VCA was also performed following the normalization step using PE-labeled beads.

## 3. Results

**Figure 2.**
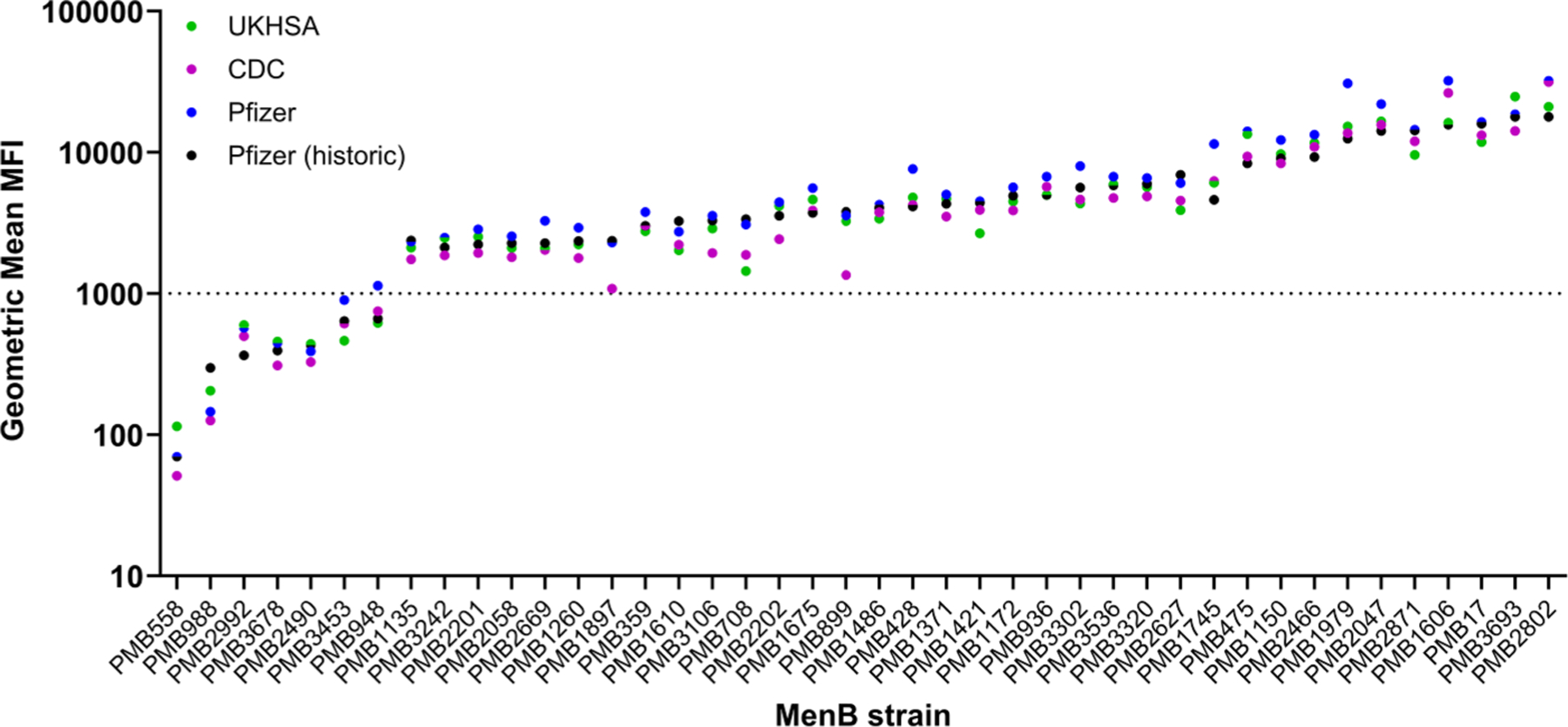
MEASURE data collected for 42 MenB strains. Geometric mean MFI values from each of the 3 laboratories as well as those collected from Pfizer approximately 2 years before the interlaboratory study (“Pfizer [historic]”) are presented for each strain. CDC = US Centers for Disease Control and Prevention; MEASURE = Meningococcal Antigen Surface Expression; MenB = meningococcal serogroup B; MFI = mean fluorescence intensity; UKHSA = UK Health Security Agency.

Factor H binding protein surface expression for each of the 42 MenB strains was determined across the 3 laboratories using the MEASURE assay. The geometric mean MFI values for each of the laboratories along with MFI values collected by Pfizer 2 years before the interlaboratory study are presented in Fig. 2.

Clustering of MFI data from the Pfizer, CDC, and UKHSA laboratories into levels either ≤1000 or >1000 demonstrated general consistency across laboratories. Statistical 2 × 2 table assessments for each of the potential pairwise comparisons are presented in Table 2. Only 1 of the 42 pairwise comparisons differed when comparing assay results from the Pfizer laboratory and either the CDC or UKHSA laboratories, amounting to 97.6 % agreement. The discrepancy for each interlaboratory comparison was attributed to strain PMB948, for which the geometric mean MFI was 1134 (ie, slightly above the breakpoint of 1000 MFI) when measured at the Pfizer laboratory but was 749 and 620 when measured at the CDC and UKHSA laboratories, respectively. All 42 pairwise comparisons of data determined from assays performed at the CDC and UKHSA were in agreement.

Intermediate precision of the MEASURE assay evaluated at each of the 3 laboratories was determined by VCA (Table 3). Precision calculations showed total %RSD values of 11.9 % (UKHSA), 15.9 % (CDC), and 20.2 % (Pfizer); thus, the precision acceptance criterion of total % RSD ≤30 % was met for all 3 laboratories. Analysis of the normalized MFI values obtained using PE beads showed that normalization had little effect on these variables, with VCA determining total %RSD values of 11.7 % (UKHSA), 15.6 % (CDC), and 23.1 % (Pfizer; Supplemental Table S1).

## 4. Discussion

The flow-cytometry–based MEASURE assay uses intact MenB bacteria to evaluate expression of fHbp in its native configuration on the cell surface in the presence of other macromolecules, such as capsular polysaccharide; this approach mimics conditions encountered by the immune system during infection [18]. The same culture conditions are used to prepare isolates for the hSBA assay employed for the clinical assessment of MenB-fHbp [15,27,31]. The broadly cross-reactive mAb, MN86–994-11–1, permits evaluation of MenB isolates in the MEASURE assay regardless of fHbp subfamily or variant [18]. Furthermore, previous investigations confirmed this mAb exhibited high affinity binding and similar association/dissociation rates for 8 fHbp variants tested, irrespective of subfamily (A or B) [18]. Based on the correlation between fHbp expression level and probability of killing in hSBA assays using serum from MenB-fHbp–vaccinated individuals, results from the MEASURE assay can be used to estimate the breadth of MenB-fHbp coverage [18]. Here, we confirm the precision and reproducibility of MEASURE assay results from Pfizer and 2 national reference laboratories, the CDC and UKHSA.

Intermediate precision of the MEASURE assay was demonstrated by VCA analysis, in which each laboratory met the assay precision criteria of total %RSD ≤30 %. The MEASURE assay provided reproducible data, with generally low %RSD for each variance component (week or analyst, nested plate [week] or nested plate [analyst], and residual) at each laboratory. Moreover, precision was largely unaffected by a normalization step using PE beads, suggesting that data normalization is not required for data analysis. However, it is important to note that the voltage of the cytometers was adjusted if the PE bead readout had drifted too far from the 13,000 MFI target, thus providing some degree of normalization and mitigating the need for additional normalization. Notably, the MEASURE assay results were similar to those collected by Pfizer 2 years before the interlaboratory study, indicating long-term assay stability. Furthermore, the geometric mean of MFI values for each of 42 MenB strains were generally consistent across all 3 laboratories: pairwise comparisons of fHbp expression levels for all 42 MenB test strains showed an agreement level of >97 % across the 3 laboratories when strains were grouped based on geometric mean MFI levels ≤1000 or >1000. Overall, the findings of this interlaboratory study demonstrate that the MEASURE assay is robust and reproducible and, therefore, reliable for the quantification of fHbp surface expression regardless of laboratory. Moreover, the protective thresholds of fHbp expression levels that correlated with hSBA killing in a previous study [18] can be applied to MEASURE data generated across laboratories.

The 42 strains evaluated in this study represent an important study strength that is crucial for extrapolation of study findings. Collectively, the strains code for 16 subfamily A and 14 subfamily B variants that are representative of the phylogenetic diversity of fHbp. The fHbp variants expressed by these strains correspond to variants encoded by ≥79 % of invasive isolates in collections from the United States and Europe [37,38]. Clonal complex representation among the 42 MenB strains corresponds to ≥91 % of invasive isolates from these collections [37,38]. Furthermore, these 42 strains span the full range of fHbp expression, from well below the MFI breakpoint of 1000 [18] to greater than 10,000 MFI, supporting the relevance of these findings to the full breadth of invasive MenB strains from various global regions.

Many of the geometric mean MFI values obtained for the MenB test strains were clustered close to the 1000 MFI breakpoint. Nevertheless, the MEASURE assay demonstrated high agreement between the laboratories: all 42 pairwise comparisons of data from the CDC and UKHSA laboratories were in agreement, and only 1 of the 42 pairwise comparisons (PMB948) differed when comparing assay results from either the CDC or UKHSA laboratories to the Pfizer laboratory. In interpreting MEASURE assay results, it is important to note that the previous study establishing an MFI of 1000 as the cutoff for potential susceptibility to killing in the hSBA assay is conservative rather than absolute. Use of an MFI value of 500 as the cutoff provided an 87.4 % probability that an isolate with MFI exceeding this level would be killed in the hSBA assay [18]. As observed for strain PMB948 in the current study, discordant categorization of a given strain as being above or below the cutoff between laboratories should be interpreted with some degree of latitude because such discrepancy might not have occurred if a less conservative cutoff had been used.

Cross-reactivity of MenB-fHbp–induced antibodies has been previously demonstrated in clinical studies using hSBA assays with 14 MenB test strains [32]. However, strains expressing very low levels of fHbp may not be susceptible to MenB-fHbp–induced antibodies [17,18]. The MEASURE assay was developed to accurately quantify fHbp surface expression as a tool to inform breadth of coverage [17,18]. In contrast to MenB-fHbp, evaluation of MenB-4C immunogenicity has primarily relied on the use of 4 MenB indicator strains that encode for protein variants identical or closely related to the vaccine antigens and express the antigens at high levels [12,19,40]. Consequently, hSBA responses determined using these 4 indicator strains provide limited information on cross-reactivity of MenB-4C–induced antibodies to strains that encode for sequence-diverse protein variants and/or low levels of protein expression [17]. For this reason, the Meningococcal Antigen Typing System (MATS) assay was developed as a tool to help predict susceptibility of a given strain to antibodies elicited by MenB-4C based on the combined factors of protein expression levels and immunologic cross-reactivity [41]. Briefly, MATS measures the level of protein expression in a bacterial lysate using an enzyme-linked immunosorbent assay (ELISA) for each of the recombinant proteins in MenB-4C (fHbp, Neisserial adhesin A [NadA], and Neisserial heparin binding antigen [NHBA]) [41]. Immunologic cross-reactivity of MenB-4C–induced antibodies to each of the protein antigens is estimated by comparing ELISA reactivity to results from a protein-specific reference strain [41]. A MenB strain is considered MATS-positive if the resulting “relative potency” value for any of the 3 recombinant proteins exceeds the “positive bactericidal threshold” for that protein, which is derived by correlating relative potency values with killing in hSBA [41]. A MenB strain that harbors a porin A serosubtype (per phenotypic or genotypic assessment) identical to the one included in the vaccine is also considered MATS-positive [38,41].

Although both the MATS and MEASURE assays were developed to estimate breadth of coverage for MenB-fHbp and MenB-4C, respectively, it is important to note key differences between the two. The MATS ELISA measures protein in a whole cell lysate; by contrast, the MEASURE assay uses intact MenB strains to selectively probe fHbp present at the bacterial surface [18,41]. Development of each assay was based upon comparison of the primary MATS or MEASURE data with hSBA activity of vaccinated human sera for a subset of MenB strains, but using different approaches: MATS data for 57 MenB strains were compared with susceptibility of those strains in hSBA to sera pooled from toddlers vaccinated with MenB-4C [41], whereas fHbp surface expression for 109 MenB strains determined in MEASURE was compared with susceptibility of those strains in hSBA using MenB-fHbp–immune sera from young adults [18]. As the immune response to vaccination can vary as a function of age [42], enlisting the use of sera from toddlers or young adults is a variable that should not be overlooked. Additionally, predictions of strain susceptibility to MenB-fHbp from the MEASURE assay derive from the expression level of a single protein [18], whereas the MATS assay considers expression level and cross-reactivity for 3 different proteins in aggregate, as well as PorA variant, to predict strain susceptibility to MenB-4C [18,41]. It is important to note that although the respective assays can help inform which MenB isolates are likely to be susceptible to MenB-fHbp or MenB-4C immune sera, the assays are not able to predict the proportion of human subjects who produce a functional bactericidal response and, in turn, do not permit conclusions related to vaccine effectiveness [17,18,33].

A previous study evaluated interlaboratory reproducibility for the MATS assay across 7 laboratories [43]; however, there are key differences between the design of that study and the MEASURE interlaboratory study reported here. In contrast with the diverse, 42-strain panel used in this study, the MATS interlaboratory study used 17 MenB strains that collectively spanned the assay range of MATS ELISA values for the 3 MenB-4C protein antigens; furthermore, testing for each of the 3 proteins was limited to non-overlapping subsets of the 17 strains expressing the respective proteins at sufficient levels [43]. Determination of assay precision also notably differs between the two interlaboratory studies: assay precision for the current MEASURE study was calculated by pairwise analysis of fHbp expression at MFI levels of either ≤1000 or >1000 for each of the 42 test strains, whereas precision for the MATS interlaboratory study was based on quantitative measurement of relative potency values [43]. The MATS relative potency values were highly correlated across the 7 laboratories, with interlaboratory coefficients of variation for the 3 proteins ranging from 7.9 % to 16.5 %; intralaboratory coefficients of variation ranged from 19.8 % to 38.3 % [43].

This study has confirmed that the MEASURE assay is robust, with performance attributes that are consistent across 3 laboratories. As noted earlier, the MEASURE assay has been used in studies that have included prevalence-based MenB collections to illustrate that fHbp is routinely detected at the surface of disease-causing isolates at levels that are consistent with functional susceptibility to MenB-fHbp–immune sera in hSBA assays [18,33,34]. Collectively, results determined using the MEASURE assay further substantiate the selection of fHbp as a meningococcal vaccine antigen.

## Data sharing statement

Upon request, and subject to review, Pfizer will provide the data that support the findings of this study. See https://www.pfizer.com/science/clinical-trials/trial-data-and-results for more information.

All authors attest they meet the ICMJE criteria for authorship.

## Supplementary Material

Supplemental Figure

Supplemental Materials

Supplementary material associated with this article can be found, in the online version, at doi:10.1016/j.diagmicrobio.2025.116920.

## Figures and Tables

**Table 1:** MenB strains selected for the interlaboratory study.

Strain ID	fHbp variant (PubMLST peptide ID) [30]	Clonal complex	Sequence type	MEASURE geometric mean (MFI)^a^
PMB558	A40 (201)	cc11	11	70
PMB988	A20 (106)	cc269	305	297
PMB2992	A05 (45)	cc213	3635	365
PMB3678	B91 (123)	cc41/44	340	395
PMB2490	A22 (19)	cc41/44	9622	430
PMB3453	A19 (16)	cc35	35	639
PMB948	B09 (13)	NA^c^	5963	664
PMB3242	B16 (4)	cc41/44	4489	2122
PMB2201	A05 (45)	cc213	7299	2230
PMB2669	B03 (14)	cc269	925	2274
PMB2058	A12 (24)	cc269	2976	2275
PMB1260	A19 (16)	cc8	2174	2350
PMB1897	B16 (4)	cc41/44	154	2370
PMB1135^b^	B01 (55)	cc41/44	44	2372
PMB359	B16 (4)	cc41/44	41	3021
PMB1610	B08 (260)	cc41/44	44	3253
PMB3106	A42 (165)	NA^c^	5100	3290
PMB708	A07 (21)	cc162	162	3363
PMB2202	A22 (19)	cc212	212	3552
PMB1675	A12 (24)	cc41/44	2851	3721
PMB899	A47 (31)	NA^c^	7284	3796
PMB1486	B13 (54)	cc269	**17634**	4043
PMB428	A22 (19)	cc269	13323	4135
PMB1371	A17 (49)	cc32	32	4321
PMB1421	A26 (23)	cc60	6148	4395
PMB1745^b^	A05 (45)	cc213	2100	4620
PMB1172	A15 (25)	cc549	5874	4918
PMB936	B15 (252)	cc41/44	11268	4992
PMB3302	A04 (180)	cc162	162	5636
PMB3536	B03 (14)	cc41/44	41	5816
PMB3320	B09 (13)	cc269	1991	5991
PMB2627	B05 (8)	cc269	2972	6931
PMB475	B24 (1)	cc32	32	8352
PMB1150	B23 (110)	cc213	213	9076
PMB2466	B52 (35)	cc41/44	41	9258
PMB1979	B44 (15)	cc269	1195	12479
PMB2047	B44 (15)	cc269	283	14151
PMB2871	A63 (84)	cc41/44	2136	14296
PMB1606	A76 (176)	cc269	3082	15673
PMB17	B02 (87)	cc32	32	15953
PMB3693	A44 (99)	NA^c^	17631	17812
PMB2802	A22 (19)	cc41/44	43	17849

fHbp = factor H binding protein; MEASURE = Meningococcal Antigen Surface Expression; MenB = meningococcal serogroup B; MFI = mean fluorescence intensity; NA = not assigned.

a Measured at Pfizer 2 years before the interlaboratory study.

b Control strains.

c Not assigned at PubMLST.

**Table 2:** Pairwise interlaboratory comparison of the number of assays with fHbp expression at levels ≤1000 and >1000 geometric mean MFI for each of the 42 MenB strains.

Laboratory Strains	Strains with MEASURE geometric mean MFI ≤1000, n	Strains with MEASURE geometric mean MFI >1000, n	Total strains, n
	**Pfizer**		
**CDC**			
Strains with MEASURE geometric mean MFI ≤1000, n	6	1	7
Strains with MEASURE geometric mean MFI >1000, n	0	35	35
Total strains, n	6	36	42
	**UKHSA**		
**Pfizer**			
Strains with MEASURE geometric mean MFI ≤1000, n	6	0	6
Strains with MEASURE geometric mean MFI >1000, n	1	35	36
Total strains, n	7	35	42
	**CDC**		
**UKHSA**			
Strains with MEASURE geometric mean MFI ≤1000, n	7	0	7
Strains with MEASURE geometric mean MFI >1000, n	0	35	35
Total strains, n	7	35	42

CDC = US Centers for Disease Control and Prevention; fHbp = factor H binding protein; MEASURE = Meningococcal Antigen Surface Expression; MenB = meningococcal serogroup B; MFI = mean fluorescence intensity; UKHSA = UK Health Security Agency.

**Table 3:** Assessment of intermediate precision for the MEASURE assay at 3 laboratories using primary MFI values.

Laboratory	Variance component^a^	Estimated variance	Standard deviation (log_10_)	% Total variance	%RSD
**UKHSA**	Week	0.0000	0.0000	0.00	0.0
	Nested plate [week]	0.0001	0.0120	5.42	2.8
	Residual	0.0025	0.0500	94.58	11.6
	Total	0.0026	0.0514	100.00	11.9
**CDC**	Analyst	0.0000	0.0000	0.00	0.0
	Nested plate [analyst]	0.0008	0.0276	16.21	6.4
	Residual	0.0039	0.0627	83.79	14.5
	Total	0.0047	0.0685	100.00	15.9
**Pfizer**	Analyst	0.0001	0.0122	1.97	2.8
	Nested plate [analyst]	0.0001	0.0093	1.15	2.1
	Residual	0.0073	0.0854	96.88	19.9
	Total	0.0075	0.0868	100.00	20.2

CDC = US Centers for Disease Control and Prevention; MEASURE = Meningococcal Antigen Surface Expression; MFI = mean fluorescence intensity; RSD = relative standard deviation; UKHSA = UK Health Security Agency.

a Variance components were based on a single analyst performing 2 assay runs, each including 4 plates, on separate weeks at the UKHSA laboratory, whereas each of the 2 assay runs was performed by an independent analyst at each of the CDC and Pfizer laboratories (Supplementary Figure S1).

## Abbreviations

CDC: US Centers for Disease Control and Prevention
ELISA: enzyme-linked immunosorbent assay
fHbp: factor H binding protein
hSBA: serum bactericidal antibody using human complement
IMD: invasive meningococcal disease
mAb: monoclonal antibody
MATS: Meningococcal Antigen Typing System
MEASURE: Meningococcal Antigen Surface Expression
MenACWY: meningococcal serogroups A, C, W, and Y
MenB: meningococcal serogroup B
MFI: mean fluorescence intensity
NadA: Neisserial adhesin A
NHBA: Neisserial heparin binding antigen
PE: phycoerythrin
RSD: relative standard deviation
SBA: serum bactericidal antibody
UKHSA: UK Health Security Agency
VCA: variance components analysis
