# Supplementary material for: Interlaboratory study to assess precision and reproducibility of the meningococcal antigen surface expression (MEASURE) assay to quantify factor H binding protein expression at the surface of meningococcal serogroup B strains
Source: Diagn Microbiol Infect Dis. Author manuscript; Available in PMC 2025 Jul 21. (PMC12278967; doi:10.1016/j.diagmicrobio.2025.116920)
Supplement: Supplemental Figure [file NIHMS2091779-supplement-Supplemental_Figure.docx]

**Supplemental Table S1. Assessment of intermediate precision for the MEASURE assay at 3 laboratories using PE bead–normalized MFI values**

| Laboratory | Variance Component^a^ | Estimated Variance | Standard Deviation (log_10_) | % Total Variance | % RSD |
| --- | --- | --- | --- | --- | --- |
| **UKHSA** | Nested plate [week] | 0.0001 | 0.0081 | 2.57 | 1.9 |
|  | Week | 0.0000 | 0.0000 | 0.00 | 0.0 |
|  | Residual | 0.0025 | 0.0500 | 97.43 | 11.6 |
|  | Total | 0.0026 | 0.0507 | 100.00 | 11.7 |
| **CDC** | Analyst | 0.0000 | 0.0000 | 0.00 | 0.0 |
|  | Nested plate [analyst] | 0.0007 | 0.0258 | 14.64 | 6.0 |
|  | Residual | 0.0039 | 0.0624 | 85.36 | 14.4 |
|  | Total | 0.0046 | 0.0675 | 100.00 | 15.6 |
| **Pfizer** | Analyst | 0.0001 | 0.0110 | 1.24 | 2.5 |
|  | Nested plate [analyst] | 0.0000 | 0.0000 | 0.00 | 0.0 |
|  | Residual | 0.0097 | 0.0983 | 98.76 | 22.9 |
|  | Total | 0.0098 | 0.0989 | 100.00 | 23.1 |

CDC=US Centers for Disease Control and Prevention; MEASURE=Meningococcal Antigen Surface Expression; PE=phycoerythrin; MFI=mean fluorescence intensity; RSD=relative standard deviation; UKHSA=UK Health Security Agency.

^a^Variance components were based on a single analyst performing 2 assay runs, each including 4 plates, on separate weeks at the UKHSA laboratory, whereas each of the 2 assay runs was performed by an independent analyst at each of the CDC and Pfizer laboratories (**Supplementary Figure S1**).
