# Supplementary material for: Interlaboratory study to assess precision and reproducibility of the meningococcal antigen surface expression (MEASURE) assay to quantify factor H binding protein expression at the surface of meningococcal serogroup B strains
Source: Diagn Microbiol Infect Dis. Author manuscript; Available in PMC 2025 Jul 21. (PMC12278967; doi:10.1016/j.diagmicrobio.2025.116920)

# Supplemental Material

## **Supplemental Figure S1. Interlaboratory study procedure performed at (A) Pfizer, (B) CDC, and (C) UKHSA.**

Adapted with permission from Loschko J et al. *Methods Mol Biol* 2019;1969:217-36.

CDC=US Centers for Disease Control and Prevention; MEASURE=Meningococcal Antigen Surface Expression; PFA=paraformaldehyde; UKHSA=UK Health Security Agency.

1. **Pfizer**


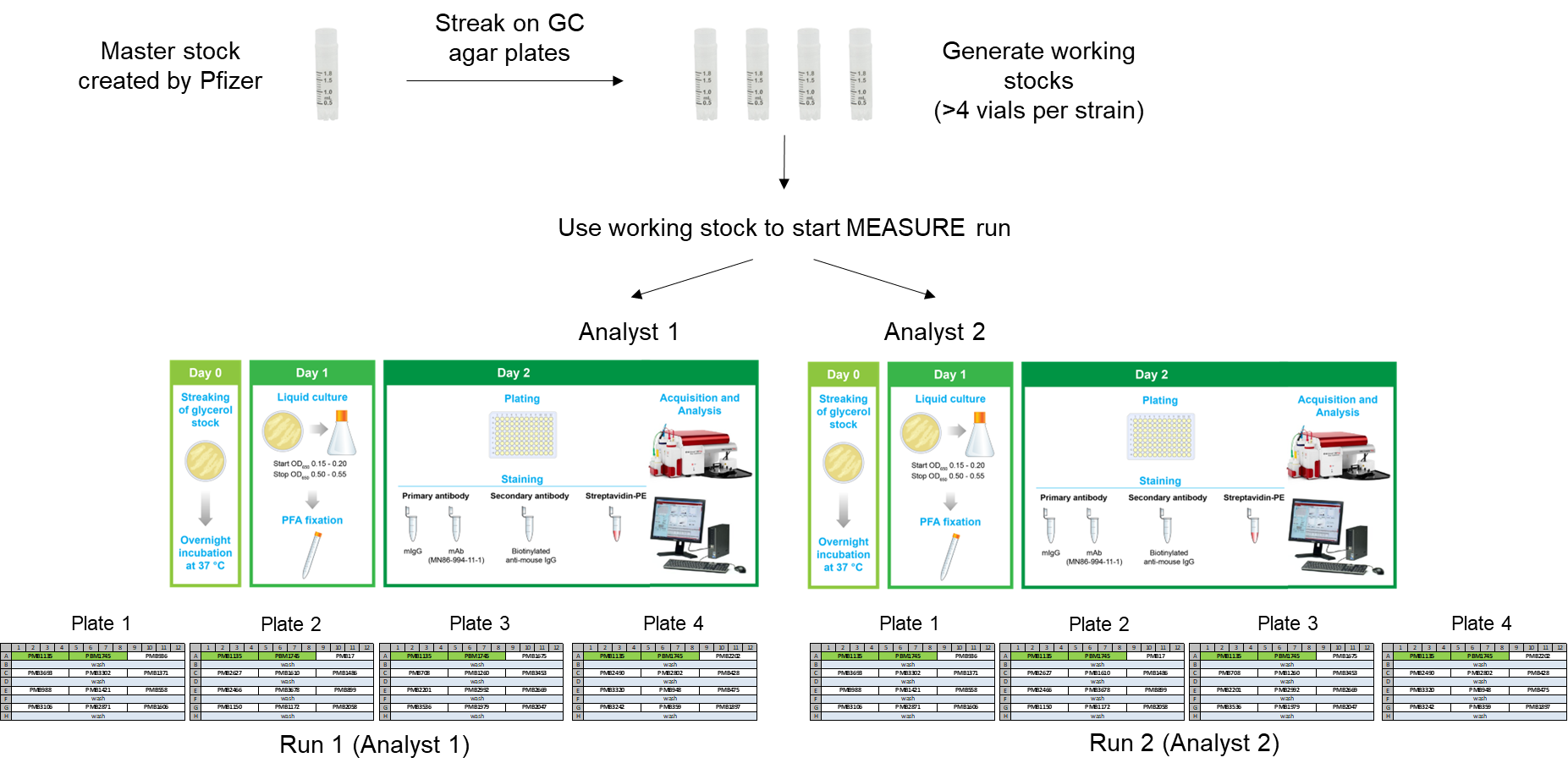


1. **CDC**


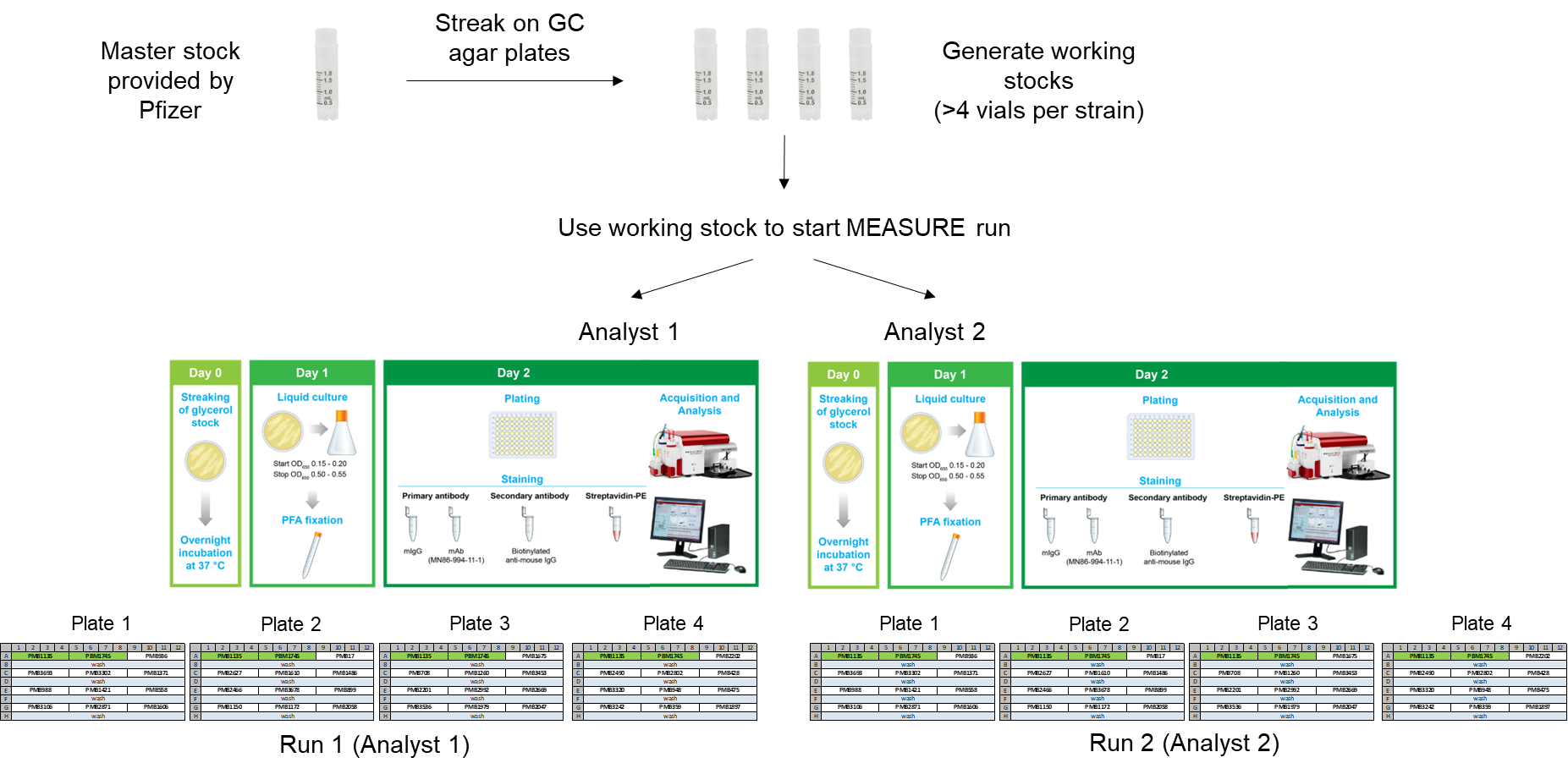


1. **UKHSA**


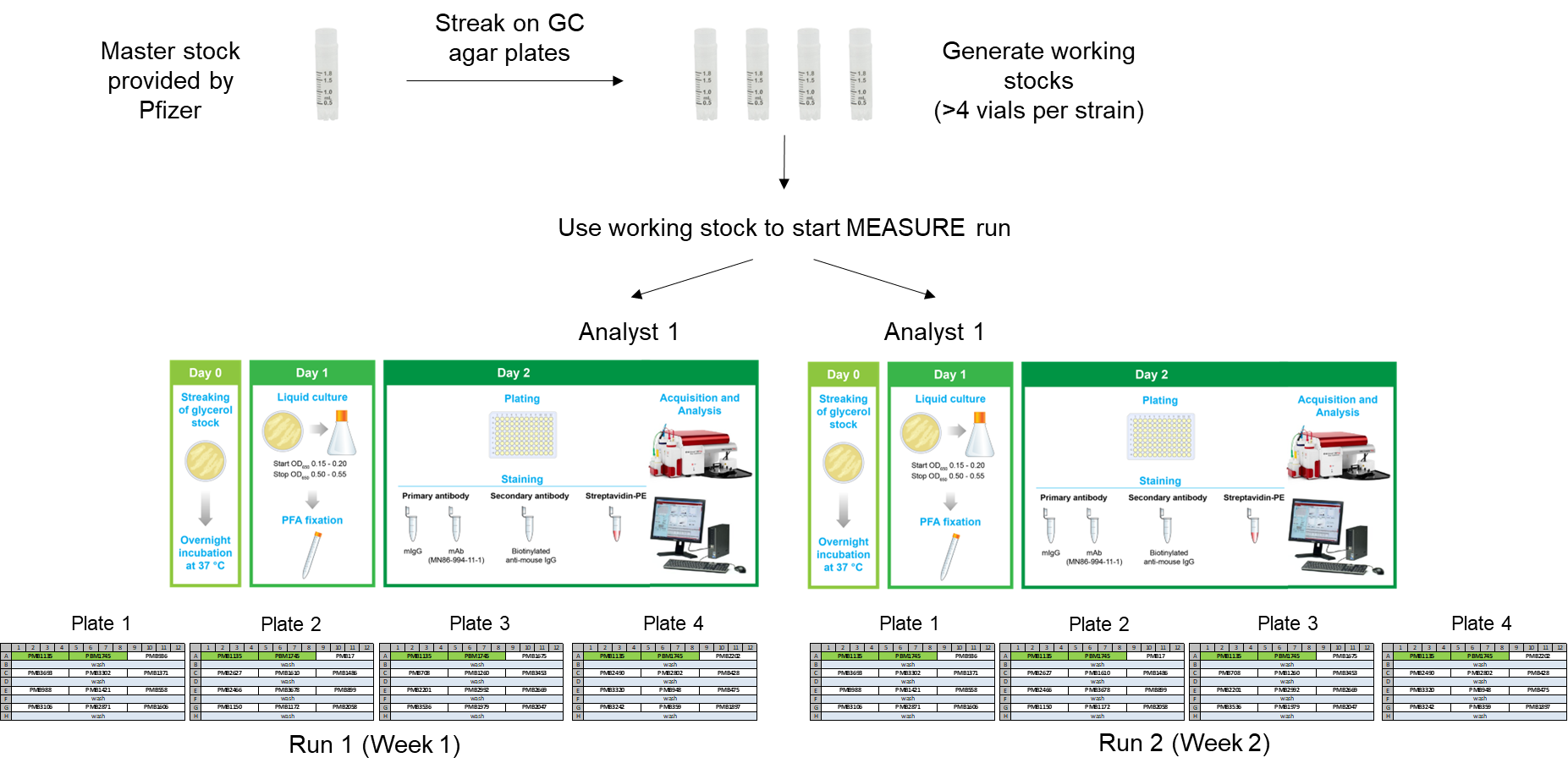

Supplement: Supplemental Materials [file NIHMS2091779-supplement-Supplemental_Materials.docx]
